# Enhancing the Yield of *Pleurotus ostreatus* Through the Addition of Nucleotides and Nucleosides

**DOI:** 10.3390/jof11070537

**Published:** 2025-07-18

**Authors:** Chenmin Tang, Yixuan Gao, Zhiguo An, Abdul Qadeer Sajid, Hanjie Ying, Zhenyu Wang, Dong Liu

**Affiliations:** 1 State Key Laboratory of Materials-Oriented Chemical Engineering, College of Biotechnology and Pharmaceutical Engineering, Nanjing Tech University, No. 30, Puzhu South Road, Nanjing 211816, China; 2 School of Chemical Engineering, Zhengzhou University, No. 100, Science Avenue, Zhengzhou 450001, China

**Keywords:** *Pleurotus ostreatus*, nucleotide, nucleoside, yield, nutritional value, enzyme activity

## Abstract

*Pleurotus ostreatus* is a mushroom species renowned for its abundant nutritional and medicinal properties. Nevertheless, the yield of its fruiting bodies has long remained at a standstill, making it arduous to achieve substantial improvements. Because the traditional composting approach for enhancing the yield of Pleurotus ostreatus has drawbacks such as a long duration and a high susceptibility to mold contamination, incorporating nutritional supplements into the culture medium of *P. ostreatus* has emerged as a relatively straightforward yet effective approach to enhancing its yield. This study was predicated on the roles of nucleotides and nucleosides in cellular metabolism and signal transduction. These substances were applied during the cultivation process of *P. ostreatus* to investigate their impact on the growth and nutritional composition of this mushroom. The findings of this study demonstrate that the supplementation of nucleotides and nucleosides not only improved the yield and biological efficiency of *P. ostreatus* but also increased its dietary fiber content and amino acids. Furthermore, this research has disclosed that nucleotides and nucleosides exert a notable influence on the lignocellulolytic enzyme system. This investigation provides a scientific foundation for the development of novel yields—enhancing agents for *P. ostreatus* and offering new insights into cultivation techniques for the progress of *P. ostreatus* cultivation techniques in both academic and practical arenas.

## 1. Introduction

Global food shortages have emerged as a severe challenge faced by all nations. Traditional agriculture and animal husbandry, characterized by long production cycles, extensive land occupation, and high climate dependency, have struggled to meet the escalating demands from the growing population. Therefore, food derived from microorganisms has gained increasing attention.

Mushrooms, a type of macroscopic filamentous fungi, are abundant in nutrients including proteins, dietary fibers, and vitamins. Moreover, they contain secondary metabolites that possess antioxidant, immunomodulatory, and anticancer activities. They are extensively applied in the fields of food, health products, and medicine and have become crucial alternative resources for addressing food security issues [1]. *Pleurotus ostreatus*, one of the most widely cultivated mushroom species, is rich in protein and essential amino acids and possesses multiple functions such as regulating blood lipids, blood sugar, as well as enhancing immunity [2]. However, the stable structure of lignocellulose in traditional substrates makes it difficult for *P. ostreatus* to effectively utilize them, severely restricting the improvement of yield [3]. Therefore, optimizing the nutritional structure of the culture medium or adding exogenous substances that are more readily absorbed by *P. ostreatus* is anticipated to effectively facilitate its growth and increase the yield.

Presently, traditional approaches to enhance the yield of *P. ostreatus* are primarily focusing on optimizing its cultivation substrates. This is accomplished either through composting agricultural waste (such as straws and corncobs) or by sourcing substrates with higher nutritional richness, enabling *P. ostreatus* to more efficiently utilize the nutrients within the substrates and thus boost its yield [4]. Incorporating sawdust from fig trees into the cultivation substrate of *P. ostreatus* has been proven to significantly increase the dry matter, lipid, and mineral content of the mushrooms [5]. Additionally, supplementing the cultivation substrate with defatted pistachio powder and defatted almond powder has been reported to enhance the yield of *P. ostreatus* by 30% [6]. Although the composting of agricultural waste is environmentally friendly, this process is relatively time-consuming. Moreover, it is susceptible to the proliferation of other microorganisms (notably molds), which can generate harmful metabolites [7]. These by-products may exert negative effects on subsequent *P. ostreatus* cultivation. Furthermore, substrates with abundant nutrients typically come at a higher cost, posing challenges to the realization of industrial-scale production. It is also noteworthy that several studies have indicated that numerous bacteria belonging to the genera *Bacillus*, *Pseudomonas*, or *Bradyrhizobium* appear to stimulate the mycelial growth of certain cultivated mushroom species (including *Agaricus bisporus*, *P. ostreatus*, or *P. eryngii*) either in compost or in vitro [8]. Nevertheless, there are no commercially available supplements based on microorganisms that promote mushroom growth in the market [9].

To address the above-mentioned challenges, it is imperative to develop a more stable, safe, and suitable growth promoter for *P. ostreatus* cultivation. Nucleotides, as fundamental substances widely found in animals, plants, and microorganisms, play crucial roles in biological systems. Existing research predominantly focus on the effects of nucleotides on animals and plants; substantial evidence indicates that nucleotides can modulate the immune system in animals, mitigate liver damage, and improve conditions associated with neurodegenerative diseases [10]. In plants, nucleotides are involved in growth and development processes while enhancing their ability to withstand external environmental stresses [11]. Although there is currently a lack of comprehensive research on the role of nucleotides in the growth and development of *P. ostreatus*, considering that 5′-nucleotides are not only crucial flavor (umami) components [12], but also that adenosine triphosphate (ATP), one of the 5′-nucleotides, can directly act as an energy source and be involved in the life activities of mushrooms [13]. Therefore, it is reasonable to postulate that these essential life components also exert a positive influence on the growth of *P. ostreatus* and the synthesis of its metabolites. Furthermore, nucleotide phosphorylation serves as a pivotal step in both oxidative phosphorylation and substrate-level phosphorylation. It directly facilitates the storage and release of energy, thereby providing support for fungal growth, motility, and secondary metabolism [14], to further investigate whether phosphorylation is necessary. In this study, *P. ostreatus* was taken as the research object, and nucleotides and nucleosides were used as yield enhancers to explore their influences on the growth of *P. ostreatus* and the nutritional components within its fruiting bodies. The aim of this research is to develop a safer and more stable growth promoter for edible fungi, while providing novel insights and a scientific basis for traditional edible fungus cultivation practices.

## 2. Materials and Methods

### 2.1. Strains and Materials

Strains: *Pleurotus ostreatus* provided by Kunming Edible Fungi Research Institute of All-China Federation of Supply and Marketing Cooperatives, were cultivated on potato dextrose agar (PDA) slants.

### 2.2. Medium and Culture Conditions

The culture medium contained soybean flour (30.0 g/L), sucrose (10.0 g/L), KH_2_PO_4_ (3.0 g/L), and MgSO_4_ (1.5 g/L), without adjusting the pH. The medium was sterilized at 121 °C for 30 min and then prepared for use. For the seed culture, three loops of pea size from a slant culture were inoculated into a 250 mL flask with 100 mL of seed medium and cultivated for 10 days in a reciprocal shaker at 140 rpm and 25 °C.

The cultivation medium comprises sawdust of miscellaneous wood (60%), cottonseed hulls (20%), wheat bran (17%), lime (1%), and gypsum (1%). The sawdust and cottonseed hulls need to be presoaked, and after the mixture, the moisture content is approximately 60%. Different proportions of nucleosides (Merck (Burlington, MA, USA), ES-008) and nucleotides (Merck (Burlington, MA, USA), N2635) need to be added to each experimental group, and the specific additional amounts are shown in Table 1 and Table 2. After thorough mixing, the mixture was filled into polyethylene sealed bags (20.0 cm × 30.0 cm) at approximately 1.0 kg per bag, with six independent replicates per group. The filled bags were sterilized at 121 °C for 3–4 h. Once the temperature of the cultivation materials dropped below 25 °C, liquid spawns were inoculated at a rate of 10% of the dry weight of the raw materials. Subsequently, they were cultured at 25 °C with a relative air humidity of 70% for approximately 45 d.

### 2.3. Analysis Methods

#### 2.3.1. Mycelial Growth Rate and Biomass

Measurement of mycelial growth rate: The strain was inoculated into the glass test tubes (100 mL) filled with the cultivation raw materials. The length of mycelial growth was measured every day, with five independent replicates per group.

Determination of biomass: The method of hydrolyzing chitin from fungi into *N*-acetyl glucosamine was referred to and slightly adjusted [15]. Specifically, 0.5 g of each group of samples was obtained after freeze-drying and added to 5.0 mL of 72% H_2_SO_4_ (Sinopharm Chemical Reagent Co., Ltd. (Shanghai, China) CAS: 7664-93-9) for a reaction at 130 rpm and 25 °C for 30 min. Subsequently, 54.0 mL of distilled water was added, and the reaction was carried out at 121 °C for 2 h. The pH was adjusted to 7.0 with NaOH for measurement. A total of 3.0 mL of each group of samples was taken and mixed with 5% NaNO_2_ and 5% KHSO_4_, with shaking and uniform mixing for 15 min. After centrifugation of the samples, the supernatant was taken and mixed with an appropriate amount of NH_4_SO_3_NH_2_ (Sinopharm Chemical Reagent Co., Ltd. (Shanghai, China) CAS: 7773-06-0) for 5 min; then, an appropriate amount of 0.5% 3-methyl-2-benzothiazolone hydrazide hydrochloride was added and mixed uniformly for 1 min. The samples were placed in a boiling water bath for 3 min. After the reaction solution cooled, an appropriate amount of 0.5% FeCl_3_ was added and left to stand for 30 min. Finally, the absorbance was detected at 650 nm.

#### 2.3.2. Yield and Biological Efficiency of Mushroom

Assessment of the yield and biological efficiency of mushroom: After drying the mushroom at a low temperature of 50 °C, the dry weight, yield and biological efficiency were remeasured.

#### 2.3.3. Components of Lignocellulose

The determination of the components of lignocellulose was conducted with slight modifications based on the method proposed by Morais et al. [16]. Briefly, for each group of samples, 0.3 g was precisely weighed, and 3.0 mL of 72% H_2_SO_4_ was added. The mixture was subjected to oscillation reaction at 30 °C for 1 h. Upon the completion of the reaction, 84 mL of distilled water was added, and the mixture was placed in a sterilizer at 121 °C for 1.5 h. The reaction liquid was subjected to suction filtration. The filter residue was first dried at 60 °C to a constant weight and then placed in a muffle furnace at 550 °C for 4 h. The difference in weight between the two weighing was the mass of lignin. The determination of cellulose and hemicellulose necessitated analysis by high-performance liquid chromatography (HPLC, Agilent 1290 (Agilent Technologies, Santa Clara, CA, USA)). The specific procedure was to prepare comparison samples for sugar loss under high temperature and high pressure. Then, 100 mg of glucose and 70 mg of xylose were weighed and added to 100 mL of distilled water, followed by the addition of 3.5 mL of 72% H_2_SO_4_. Each sample group and the comparison sample were placed in a sterilizer at 121 °C for 1 h. The analysis conditions of high-performance liquid chromatography were as follows: the injection volume was 10 μL, the mobile phase was 5 mmol/L sulfuric acid, which was filtered through a 0.2 μm water filter paper and degassed by ultrasound, the flow rate was 0.5 mL/min, the temperature of the chromatographic column (Agilent C18 (Agilent Technologies, Santa Clara, CA, USA), 4.6 mm × 150 mm, 5 μm) was 65 °C, the detector was a differential refractive index detector, the detector temperature was 45 °C, and the running time was 15 min.

#### 2.3.4. Enzymatic Activity

Detection of activities of cellulase, xylanase, and laccase: The preparation of the crude enzyme solution was based on the method delineated by Sandhya et al. [17]. Specifically, 1.0 g of each sample was obtained, and 5 mL of distilled water was added. The mixture was shaken and homogenized at 30 °C and 180 rpm for 30 min. Subsequently, it was centrifuged at 10,000 rpm for 10 min, and the supernatant was set aside for subsequent use. The determination of enzyme activity was conducted using an enzyme activity assay kit (Macklin Biochemical Technology Co., Ltd. (Shanghai, China) C805911, X805921, L930375), with three parallels for each sample.

#### 2.3.5. Nutritional Evaluation and Heavy Metals

The determination of crude polysaccharides was conducted using the phenol–sulfuric acid method [18]. The specific procedure for sample treatment is as follows: Weigh 0.2 g of the sample and add it to 20 mL of distilled water. Keep it at 4 °C overnight and then subject it to a reaction at 140 °C for 2 h. Heat the reaction liquid to 100 °C until the liquid is essentially non-flowable. Add 5 mL of distilled water and mix well. Subsequently, add 75 mL of absolute ethanol and mix for 30 s. Once again, keep it at 4 °C overnight. After centrifugation, remove the supernatant. Subsequently, 3.0 mL of concentrated H_2_SO_4_ was added, and the mixture was shaken uniformly. The reaction solution was placed in a water bath at 100 °C for 15 min, rapidly cooled with cold water, and the absorbance was detected at 490 nm.

The determination methods of heavy metal ions lead and cadmium referred to Liu et al. [19], with slight modifications. Specifically, after drying the fruiting bodies of *P. ostreatus*, they were ground and sieved. For each sample, 0.5 g was taken, and 5 mL of HNO_3_ was added. The samples were digested using a microwave digestion apparatus until the reaction solution turned transparent. Once the reaction solution cooled, it was heated on an electric stove and concentrated to 3.0 mL. Subsequently, it was atomized in a graphite furnace (Tianmei (China) Scientific Instruments Co., Ltd. (Zhongshan, China) GF-30), and the absorbance was measured at 228.8 nm.

Dietary fiber was determined by the methods of AOAC 991.43 [20]. Before the determination, the samples were required to be defatted three times with petroleum ether (25 mL/g) and desugared three times with 85% ethanol (10 mL/g) [21].

Protein was determined by Guan et al. [22]. In brief, an appropriate amount of sample was taken and 50 mL of NaOH was added. The crude protein was extracted with the assistance of ultrasonic treatment at 50 °C for 2 h. After the reaction, the supernatant was collected by centrifugation, the pH value was adjusted to neutral, and the content of protein was detected using the Kjeldahl nitrogen determination method (Shandong Green Kayray Precision Instruments Co., Ltd. (Heze, China) GK-600) [23].

The determination of amino acids was conducted by employing the method of Singh and Sharma [24]. Concretely, weigh the sample precisely at 1.0 g, digest the sample using 1 mL of sodium carbonate buffer solution (pH = 9.0) and 200 μL of a 3% acetonitrile solution of 2,4-dinitrochlorobenzene derivative. The sample was filtered using a 0.22 μm filter membrane and analyzed by HPLC (Agilent 1290 (Agilent Technologies, Santa Clara, CA, USA)). The HPLC conditions referred to those utilized by Feng et al. [25].

The detection method for nucleotides refers to Garcia et al. [26]. In a nutshell, 0.2 g of each sample group was weighed out, 4 mL of distilled water was added, and ultrasonic extraction was conducted for 40 min. After standing for 20 min, the mixture was passed through a 0.22 μm filter membrane and analyzed by HPLC (Agilent 1290). The mobile phase consisted of methanol and 10 mM ammonium acetate solution, with a flow rate of 0.8 mL/min and a column temperature of 30 °C.

### 2.4. Statistical Analysis

The growth rate of mushroom mycelium, mycelial biomass, biological efficiency, nutritional components and enzyme activity were conducted using ANOVA with Tukey HSD post hoc analysis using Origin pro-2023 software and SPSS 26.0 software. All analyses were carried out in triplicate, and the results were expressed as the mean ± standard deviation (SD). Duncan’s multiple-range test was used for multiple comparisons. A *p* value of 0.05 was considered to be significant.

## 3. Results

### 3.1. The Mycelium Growth Rate

**Figure 1:**
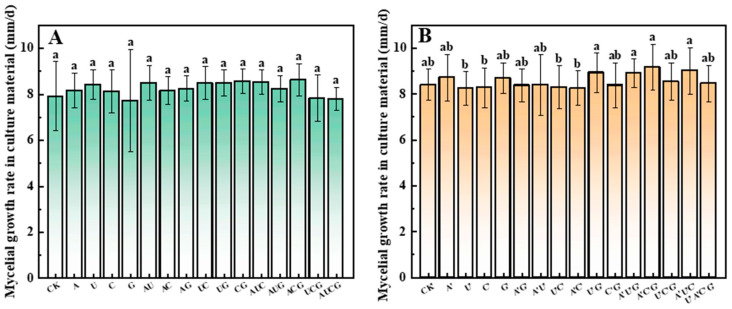
The growth rate of *P. ostreatus* mycelium within the cultivation substrate. (**A**) Nucleotide group. (**B**) Nucleoside group. Treatments with different letter labels indicate statistically significant differences between groups (*p* < 0.05), based on one-way ANOVA followed by Tukey’s post hoc test (*n* = 3).

The liquid strain was inoculated into glass test tubes filled with the cultivation substrate until the entire tube was colonized. The distance of mycelial growth was measured daily, and the average growth rate of mycelium in each group of samples was compared. The results have been shown in Figure 1. After the addition of nucleotides and nucleosides, no significant differences were exhibited in the average growth rate of mycelium in each group. This finding suggests that the addition of nucleotides and nucleosides maybe did not exert a notable effect on the growth rate of mycelium. Since the mycelium grows within the cultivation substrates, the growth status of the mycelium within the cultivation substrates cannot be effectively detected through visual observation. Moreover, although the growth rates of the mycelium are similar, the compactness of the mycelium cannot be clearly observed. Hence, the growth rate of the mycelium is merely regarded as a reference. It is also necessary to conduct the detection of the mycelial biomass to ensure that the differences in the mycelium growth among the groups of samples can be identified.

### 3.2. Analysis of Biomass

**Figure 2:**
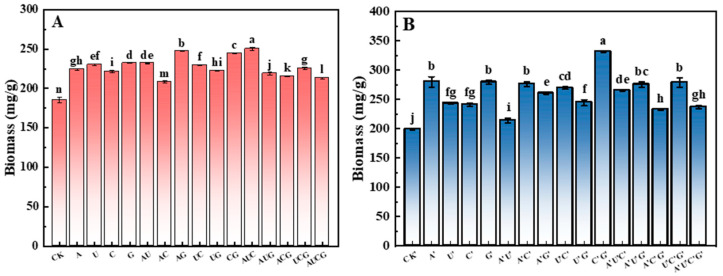
The mycelial biomass of *P. ostreatus* in cultivation substrates under different treatment conditions. (**A**) Nucleotide-supplemented groups. (**B**) Nucleoside-supplemented groups. Mycelial biomass was expressed in mg/g dry weight. Treatments with different letter labels indicate statistically significant differences between groups (*p* < 0.05), based on one-way ANOVA followed by Tukey’s post hoc test (*n* = 3).

Further detection of the mycelial biomass in each group revealed that the mycelial biomass in the experimental groups with nucleotides added was significantly higher than that in the control group (Figure 2). Notably, the biomass increments in the AU and AUC groups were the most prominent, attaining 247.39 mg/g and 249.96 mg/g, respectively, representing increases of 33.62% and 35.01% compared to the control group. Similarly, the mycelial biomass in the experimental groups supplemented with nucleosides also exhibited significant elevations relative to the control group. Among them, the C’G’ group boasted the highest biomass of 331.81 mg/g, which was 66.48% higher than that of the control group. This outcome indicates that although no discernible differences in the growth rate of mycelia were visually detectable among the groups, there were rather significant distinctions in their biomass. Both nucleotides and nucleosides can effectively enhance the biomass of *P. ostreatus* mycelia. Overall, the biomass of the groups with nucleosides added surpassed that of the groups with nucleotides added.

### 3.3. The Total Yield and Biological Efficiency of P. ostreatus

**Figure 3:**
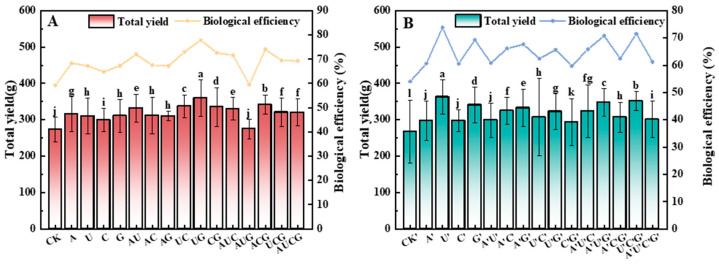
The total yield and biological efficiency in the fruiting bodies of *P. ostreatus* under different treatment conditions. (**A**) Nucleotide-supplemented groups. (**B**) Nucleoside-supplemented groups. Treatments with different letter labels indicate statistically significant differences between groups (*p* < 0.05), based on one-way ANOVA followed by Tukey’s post hoc test (*n* = 3).

The yield of mushroom serves as a crucial indicator for assessing the efficacy of yield-enhancing agents. In this study, the cultivated *P. ostreatus* gave rise to two flushes of fruiting bodies throughout the entire growth cycle. By summing up the fresh weight of the fruiting bodies from the two flushes, the total yield of *P. ostreatus* in each group was ascertained. The ratio of the total yield to the dry weight of the cultivation substrate in each bag was defined as biological efficiency. Figure 3 presents the outcomes of the total yield of *P. ostreatus* fruiting bodies and the biological efficiency in each experimental group. Following the supplementation of nucleotides and nucleosides, the yield of *P. ostreatus* fruiting bodies exhibited a significant increase. Moreover, notable differences were observed among the samples. Furthermore, the trend of biological efficiency was largely consistent with that of the total yield. In particular, in the nucleotide group, the total yield and biological efficiency of *P. ostreatus* in the UG group reached 359.2 g and 77.8%, respectively, representing an increase of 31.5% and 18.6% compared to the control group; in the nucleoside group, the total yield and biological efficiency of *P. ostreatus* in the U’ group were 362.6 g and 73.77%, respectively, which were 35.3% and 19.77% higher than those in the control group. These results suggest that the addition of nucleotides and nucleosides can effectively enhance the yield of *P. ostreatus* fruiting bodies and the biological efficiency of the cultivation substrate.

### 3.4. The Morphology of the Fruiting Body

**Figure 4:**
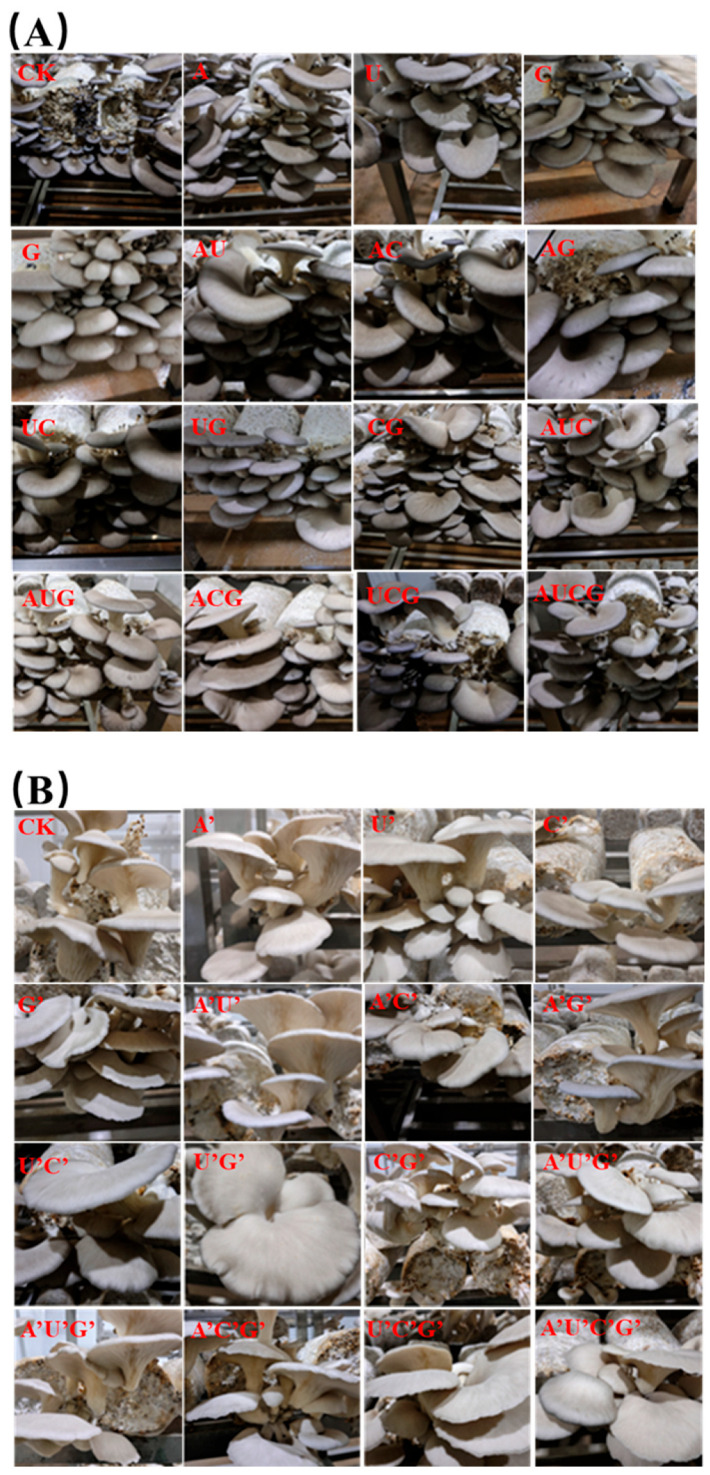
Morphology of the fruiting body of *P. ostreatus* under different treatment conditions. (**A**) Nucleotide-supplemented groups. (**B**) Nucleoside-supplemented groups.

In this study, the morphology of the fruiting bodies of *P. ostreatus* after cultivation with the addition of nucleotides and nucleosides was documented. The results are presented in Figure 4. On the whole, the treatment groups supplemented with nucleotides and nucleosides demonstrated no significant discrepancies in the morphological characteristics of the fruiting bodies in comparison with the control group, suggesting that these two types of substances do not exert adverse impacts on the normal morphological development of *P. ostreatus*. Furthermore, no massive occurrence of malformed fruiting bodies was observed in any of the treatment groups. The morphology of the fruiting bodies maintained excellent uniformity and integrity, showcasing outstanding commercial traits. Significantly, nucleotides and nucleosides augmented the yield without sacrificing the quality of the fruiting bodies, indicating that they did not disrupt the normal development process of *P. ostreatus* during the regulation of biomass accumulation.

### 3.5. The Nutrients in the Fruiting Bodies of P. ostreatus

#### 3.5.1. Dietary Fiber

**Figure 5:**
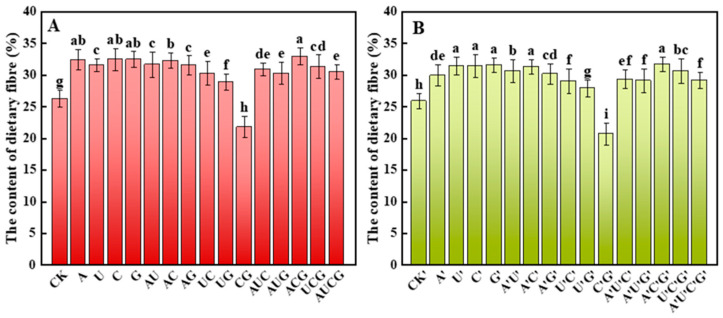
Dietary fiber content in the fruiting bodies of *P. ostreatus* under different treatment conditions. (**A**) Nucleotide-supplemented groups. (**B**) Nucleoside-supplemented groups. Dietary fiber content was expressed as a percentage of dry weight. Treatments with different letter labels indicate statistically significant differences between groups (*p* < 0.05), based on one-way ANOVA followed by Tukey’s post hoc test (*n* = 3).

Dietary fiber in mushrooms not only exhibits remarkable physicochemical properties (such as high water-holding capacity and the ability to absorb cholesterol and bile salts) but also has intervention effects on chronic diseases like obesity, metabolic syndrome, and hepatic steatosis through mechanisms like regulating the intestinal flora, improving metabolic pathways, and resisting oxidative stress [27]. The detection results of dietary fiber in the fruiting bodies of *P. ostreatus* in this study are presented as shown in Figure 5. Overall, the dietary fiber content of each sample, except for the CG and C’G’ groups, was significantly enhanced after the addition of nucleotides and nucleosides. Furthermore, the variation trends of dietary fiber content in the two experimental groups were largely consistent. The dietary fiber contents in ACG and A’C’G’ were the highest among all the groups, being 32.98% and 31.71%, respectively, which were 25.65% and 22.4% higher than those of their respective control groups. Therefore, the addition of nucleotides and nucleosides exerted a remarkable promoting effect on the dietary fiber content in the fruiting bodies of *P. ostreatus*.

#### 3.5.2. Protein

**Figure 6:**
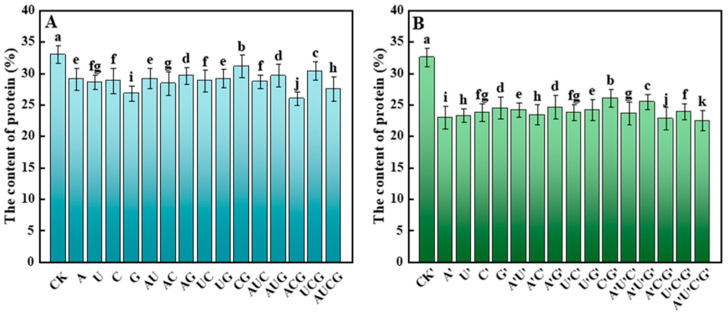
Total protein content in the fruiting bodies of *P. ostreatus* under different treatment conditions. (**A**) Nucleotide-supplemented groups. (**B**) Nucleoside-supplemented groups. Total protein was expressed as a percentage of dry weight. Treatments with different letter labels indicate statistically significant differences between groups (*p* < 0.05), based on one-way ANOVA followed by Tukey’s post hoc test (*n* = 3).

The fruiting bodies of mushrooms are rich in protein, not only with a balanced amino acid composition and high nutritional value but also contain various functional proteins with biological activity. These proteins exhibit multiple physiological activities, such as antioxidant, antitumor, immune regulation, antibacterial, and blood pressure lowering effects [28]. The detection results of the protein content in the fruiting bodies of *P. ostreatus* in this study have been shown in Figure 6. The experimental results indicate that after the addition of nucleotides and nucleosides, the protein content in the fruiting bodies of *P. ostreatus* decreased significantly, and the reduction in the protein content was more severe in the nucleoside group.

#### 3.5.3. Amino Acid

**Figure 7:**
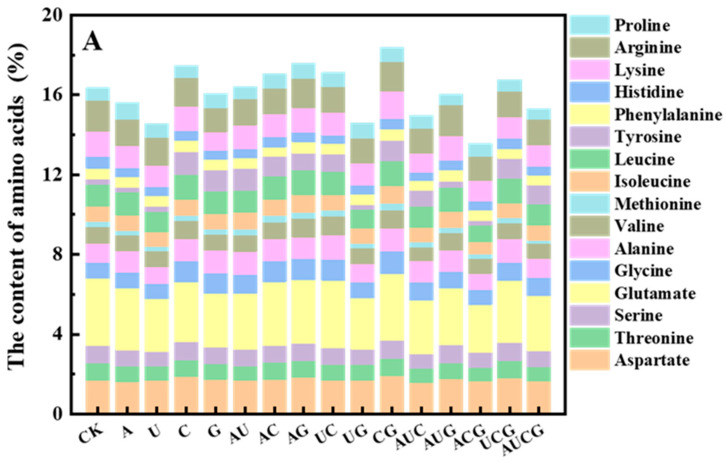

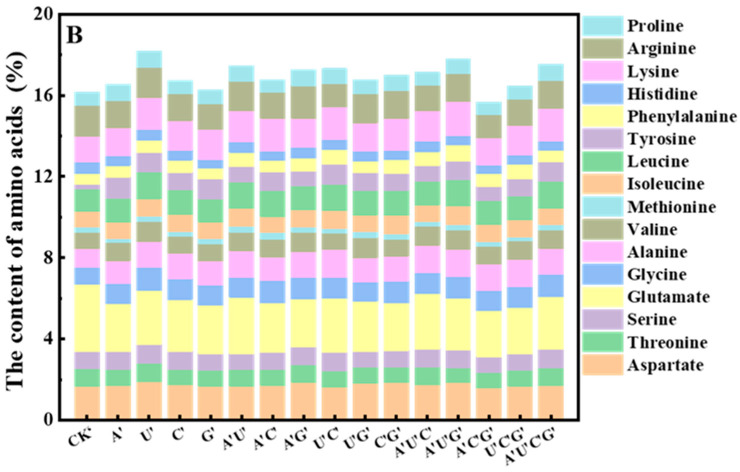
Amino acid composition in the fruiting bodies of *P. ostreatus* under different treatment conditions. (**A**) Nucleotide-supplemented groups. (**B**) Nucleoside-supplemented groups.

Although the protein content of the *P. ostreatus* fruiting bodies decreased to varying extents, further analysis revealed that the diversity of amino acids in each treatment group remained high, with their proportions being consistent (Figure 7). Specifically, within the nucleotide-supplemented group, substantial differences were detected among various samples. Among them, the CG group demonstrated the most remarkable increase in amino acid content, reaching 18.4%, which is 2.0% higher than the control group. In contrast, the nucleoside-treated group exhibited an overall increase in amino acid content, with the U’ group showing the highest level at 18.2%, an increase of 2.05% compared to the control. Notably, the total amino acid composition across all experimental groups remained highly consistent with the control group. Glutamic acid (12.8–13.2%) and aspartic acid (9.5–9.8%) were found to be the predominant amino acids in the fruiting bodies, contributing to the unique umami flavor of *P. ostreatus*. Thus, although the supplementation of nucleotides and nucleosides did not effectively enhance the protein content of *P. ostreatus*, it exerted a certain promotional effect on amino acids, with a more pronounced effect observed for nucleosides.

#### 3.5.4. Polysaccharides

**Figure 8:**
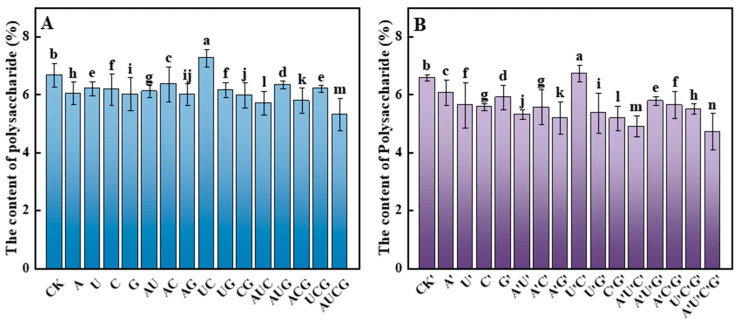
Total polysaccharide content in the fruiting bodies of *P. ostreatus* under different treatment conditions. (**A**) Nucleotide-supplemented groups. (**B**) Nucleoside-supplemented groups. Polysaccharide content was determined using the phenol–sulfuric acid and expressed as a percentage of dry weight. Treatments with different letter labels indicate statistically significant differences between groups (*p* < 0.05), based on one-way ANOVA followed by Tukey’s post hoc test (*n* = 3).

Polysaccharides originating from mushrooms represent one of their primary active constituents. Mushroom polysaccharides exhibit remarkable antioxidants, antitumor, and intestinal microbiota-regulating properties [29]. In this study, the polysaccharides in the fruiting bodies of *P. ostreatus* had been analyzed (Figure 8). Following the supplementation of nucleotides and nucleosides, the polysaccharide content across almost all samples exhibited a downward trend. Notably, within the treatment groups, the polysaccharide content in the UC and U’C’ subgroups demonstrated a slight increase, reaching 7.27% and 6.74%, respectively. These increases represented an 8.8% and 2.3% elevation compared to their respective control groups.

#### 3.5.5. Heavy Metal Ions

**Figure 9:**
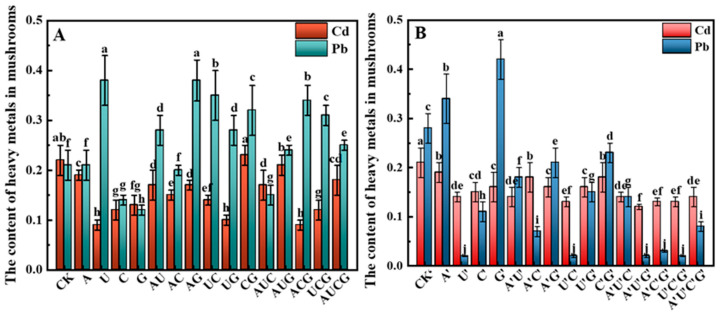
Pb and Cd contents in the fruiting bodies of *P. ostreatus* under different treatment conditions. (**A**) Nucleotide-supplemented groups. (**B**) Nucleoside-supplemented groups. Pb: lead; Cd: cadmium. Treatments with different letter labels indicate statistically significant differences between groups (*p* < 0.05), based on one-way ANOVA followed by Tukey’s post hoc test (*n* = 3).

During the process of mushroom cultivation, the phenomenon of heavy metal enrichment constitutes an issue that cannot be disregarded. A subset of mushrooms is capable of absorbing and accumulating multiple heavy metal elements from the environment, such as lead (Pb), cadmium (Cd), mercury (Hg), and so on [30]. Therefore, in this study, Pb and Cd in the fruiting bodies of *P. ostreatus* were examined, and the results presented as shown in Figure 9. After the addition of nucleotides, the Cd content in the fruiting bodies of *P. ostreatus* decreased in comparison to the control group, but the Pb content displayed distinct differences among the groups. Apart from the C, G, and AUG treatment groups where the Pb content decreased significantly, Pb was significantly enriched in the remaining groups, and the Pb content in some treatment groups exceeded the food safety standard (0.3 mg/kg). In the nucleoside group, except for the A’ and G’ groups where the Pb content increased conspicuously, both Cd and Pb in the other experimental groups demonstrated a marked decreasing trend. The degree of reduction in Cd content was relatively small, but the Pb content in the U’, U’C’, A’U’G’, A’C’G’, and U’C’G’ groups decreased extremely significantly, with only trace amounts of Pb detected. Consequently, the majority of the groups of *P. ostreatus* fruiting bodies met the food safety standards after the addition of nucleosides. Compared to nucleotides, nucleosides are more beneficial in preventing the enrichment of heavy metals in *P. ostreatus* and guaranteeing its quality.

### 3.6. Analysis of Enzyme Activity at Various Growth Phases of P. ostreatus

**Figure 10:**
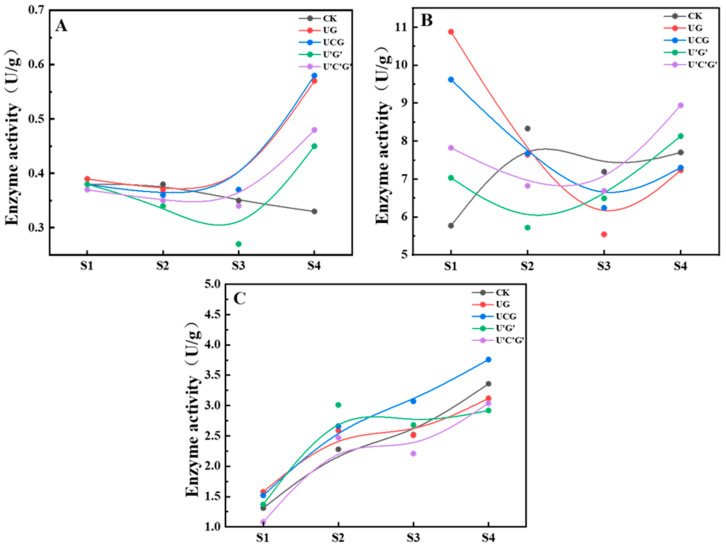
Enzyme activity analysis at different growth stages of *P. ostreatus* under different treatment conditions. (**A**) Laccase; (**B**) cellulase; (**C**) xylanase. S1: Initial mycelial growth stage; S2: Mycelium fully colonizes the substrate; S3: Primordium stage; S4: Fruiting stage.

To further investigate the mechanisms by which nucleotides and nucleosides enhance the yield of *P. ostreatus* fruiting bodies, subsequent experiments focused on experimental groups with relatively high total fruiting body yield and nutrient content (UG, UCH, U’G’, U’C’G’). A systematic analysis was conducted on the key enzyme activities at different stages, emphasizing changes in the activities of laccase, cellulase, and xylanase. The experimental results are presented in Figure 10.

During the early mycelial growth to primordium formation stage (S1–S3) of *P. ostreatus*, the lack of activity exhibited a slight overall decline trend with minimal differences among groups (Figure 10A). However, during the S3–S4 period, the lack of activity in all groups increased significantly, far exceeding that of the control group. Notably, the UCG and U’C’G’ groups demonstrated the highest laccase activity, reaching 0.57 U and 0.58 U, respectively.

Cellulase comprises endoglucanase, exoglycanase, and β-glucosidase, responsible for breaking down cellulose into absorbable glucose units. During the S1–S3 period, cellulase activity in all experimental groups was initially significantly higher than in the control group but subsequently showed a pronounced decline trend, falling below the control group level. By the S3–S4 period, activity increased again; however, only the U’C’ and U’C’G’ groups surpassed the control group, reaching 8.13 U and 8.94 U, respectively, representing increases of 5.6% and 16.1% over the control group (Figure 10B).

Xylanase degrades the Xylan component into hemicellulose, with its activity exhibiting dynamic changes throughout the growth cycle of *P. ostreatus*. Previous studies have shown that xylanase activity is relatively low during the initial mycelial colonization stage but significantly increases during primordium formation and fruiting body development stages. Xylanase activity in each group consistently increased throughout the growth process of *P. ostreatus*; notably, only the UCG group maintained higher xylanase activity than the control group at all stages, reaching a peak of 3.76 U at S4, which was 11.9% higher than the control group (Figure 10C).

These results indicate that exogenous nucleoside substances (UG, UCH, U’G’, U’C’G’) significantly enhance the degradation efficiency of lignocellulose in the cultivation substrate by differentially regulating the dynamic activities of laccase, cellulase, and xylanase.

## 4. Discussion

In this study, we initially measured the growth rate and biomass of *P. ostreatus* mycelia. The results indicated that no distinct differences were observed in the growth rate of the mycelia. However, the biomass of the mycelia was enhanced to varying extents, and the enhancement in the nucleoside-treated group was more prominent than that in the nucleotide-treated group. Nucleotides and nucleosides, as biological signaling molecules, can activate the expression of genes related to cell wall synthesis or storage substances, causing the mycelia to thicken and branch more, thereby promoting the density of the mycelia [31]. Such structural reinforcement does not alter the linear extension rate of the mycelia but can significantly increase the mycelial quantity within a unit volume [32]. Additionally, nucleosides are more prone to enter cells through passive transport than negatively charged nucleotides, and they can be rapidly converted into active nucleotides through phosphorylation within the cells, circumventing the issue of low utilization efficiency resulting from the poor membrane permeability of nucleotides [33].

Further analysis of the yield and biological efficiency of *P. ostreatus* confirmed that the addition of nucleotides and nucleosides significantly facilitated the enhancement of both the yield and biological efficiency of *P. ostreatus*. Specifically, within the nucleotide treatment group, the UG group exhibited the highest total yield and biological efficiency, showing increases of 31.25% and 18.6%, respectively, when compared with the control group. In the nucleoside-supplemented group, the U’ group demonstrated the highest total yield and biological efficiency, registering increases of 35.3% and 19.77%, respectively, relative to the control group. As precursors of nucleic acids, the exogenous addition of nucleotides and nucleosides can alleviate the energy requirements of mycelia during the degradation of lignocellulose, provide additional nutrients for the mycelia in the early growth stage, and accelerate the accumulation of mycelia in the early phase. Given the relatively high nitrogen content in nucleotides and nucleosides, they might optimize the carbon-to-nitrogen ratio in the cultivation substrate, thereby promoting the growth of *P. ostreatus* [34]. Additionally, the supplementation of nitrogen sources can stimulate the synthesis of key enzymes such as manganese peroxidase, thereby enhancing the decomposition and utilization of cellulose in the cultivation substrate by *P. ostreatus* and ultimately boosting the yield of *P. ostreatus* fruiting bodies [35].

In addition, through an analysis of the major nutrients in *P. ostreatus*, it was found that following treatment with nucleotides and nucleosides, the dietary fiber content in all samples except those in the CG group and the C’G’ group increased significantly. Moreover, no marked difference was observed in the improvement effects between the two treatment groups. Some research has indicated that certain exogenous nucleotides (such as GMP and AMP) can activate signaling pathways related to stress or growth, like the TOR or MAPK signaling pathways, thereby regulating cellular metabolism [36]. In fungi, such activation might result in a cell wall integrity response, thereby inducing the synthesis of more β-glucan and chitin. Furthermore, as the foundation for energy and nucleic acid synthesis, nucleotides may facilitate the differentiation and maturation of fruiting bodies [36]. The development of fruiting bodies is typically accompanied by thickening and structural complication of the cell wall, thereby increasing the overall amount of dietary fiber [37]. Unexpectedly, while the protein content of *P. ostreatus* exhibited a downward trend, the amino acid content in certain treatment groups increased. Notably, the effect was more pronounced in the nucleoside-supplemented group. Exogenous nucleoside-based substances are preferentially utilized for DNA or RNA replication, thereby facilitating the augmentation of amino acids. From a metabolic regulation perspective, the observed increase in total amino acid content alongside compositional stability may result from the allosteric modulation of glutamate dehydrogenase (GDH) and aspartate aminotransferase (AST) activities by exogenous nucleotides [38]. Of particular significance is the substantial increase in the absolute content of umami amino acids in the treatment groups (glutamic acid +2.3%, aspartic acid +1.8%), potentially linked to the enhanced flux of α-ketoglutarate in the tricarboxylic acid cycle stimulated by nucleotides, given that this intermediate serves as the direct precursor for glutamic biosynthesis [39]. This finding aligns with the “overflow” theory of nitrogen metabolism in microorganisms, where excess nitrogen enters amino acid synthesis via transamination when carbon skeletons are abundant [40].

During the growth of mushrooms, mycelium secretes various lignocellulolytic enzymes to decompose complex organic matter in the substrate, thereby providing the carbon source and energy required for its own growth and development [41]. These enzymes primarily include laccase, cellulase, and xylanase, which exhibit distinct activity patterns at different growth stages and significantly influence the formation and yield of fruiting bodies [42]. The marked increase in laccase activity during the S3–S4 stage (highest in UC and UCG groups) may be attributed to nucleosides acting as induction signals for lignin-degrading genes. Nucleosides might activate copper ion response elements (e.g., ACE elements) in the laccase gene promoter or provide ATP to accelerate laccase protein synthesis [43]. The significant decline in cellulase activity during the middle growth stage of *P. ostreatus* is likely due to the mycelium prioritizing exogenous nucleosides as a rapid carbon source (nucleoside → ribose → pentose phosphate pathway for energy supply) rather than initiating the energy-intensive cellulose degradation process [44]. Notably, the full-cycle superiority of xylanase in the UCG group suggests that the unmodified triad of uridine (U), cytidine (C), and guanosine (G) might serve as signal molecules to activate the transcription of xylanase genes, thereby maintaining a relatively high enzymatic activity throughout the growth cycle [45].

Ultimately, it is our contention that nucleoside-derived substances possess substantial potential for augmenting the yield of Pleurotus ostreatus. Nevertheless, these substances have not resulted in an elevation of the protein and polysaccharide content. In subsequent research undertakings, we will contemplate incorporating other exogenous nutrients concurrently with nucleoside—based substances to explore high—quality nutritional supplements capable of optimizing both the yield and nutritional composition of *P. ostreatus*.

## 5. Conclusions

In summary, considering the multiple limitations of traditional methods for increasing the yield of *P. ostreatus*, such as long-time consumption, high susceptibility to contamination, and high costs, this study added nucleotides and nucleosides as nutrients to the cultivation raw materials of *P. ostreatus*. The experimental findings indicate that the addition of nucleotides and nucleosides not only increased the yield and biological efficiency of *P. ostreatus* but also promoted an elevation in the content of dietary fiber and amino acids within the mushrooms. Additionally, it was found that the phosphate group in nucleotides had minimal impact on the yield enhancement effect; rather, the crucial factor was attributed to the core structure of the nucleoside molecule. This research has validated the potential application of nucleotide-based substances as novel biological stimulants in *P. ostreatus* cultivation. In the future, these substances can be employed in the cultivation of various commercial mushrooms, such as *Lentinula edodes*, *Volvariella volvacea*, and *Agaricus bisporus*, to more comprehensively assess the suitability of nucleoside substances in enhancing mushroom yields. This will thereby broaden the application scope of nucleoside compounds in the agricultural domain. Collectively, this study offers novel perspectives and valuable references for the development of mushroom yield-enhancing agent products.

## Figures and Tables

**Table 1:** Combinations and ratios of nucleotides.

**Nucleotide Groups**	**A (%)**	**U (%)**	**C (%)**	**G (%)**
CK	0.000	0.000	0.000	0.000
A	0.040	0.000	0.000	0.000
U	0.000	0.040	0.000	0.000
C	0.000	0.000	0.040	0.000
G	0.000	0.000	0.000	0.040
AU	0.020	0.020	0.000	0.000
AC	0.020	0.000	0.020	0.000
AG	0.020	0.000	0.000	0.020
UC	0.000	0.020	0.020	0.000
UG	0.000	0.020	0.000	0.020
CG	0.000	0.000	0.020	0.020
AUC	0.013	0.013	0.013	0.00
AUG	0.013	0.013	0.000	0.013
ACG	0.013	0.000	0.013	0.013
UCG	0.000	0.013	0.013	0.013
AUCG	0.010	0.010	0.010	0.010

Notes: Adenosine monophosphate (A); uridine monophosphate (U); cytidine monophosphate (C); guanosine monophosphate (G). Based on economic efficacy and the previous studies of our research team, the concentration of nucleotides added in this experiment was determined as 0.04% (*w*/*v*).

**Table 2:** Combinations and ratios of nucleosides.

**Nucleoside Group**	**A’ (%)**	**U’ (%)**	**C’ (%)**	**G’ (%)**
CK	0.000	0.000	0.000	0.000
A’	0.040	0.000	0.000	0.000
U’	0.000	0.040	0.000	0.000
C’	0.000	0.000	0.040	0.000
G’	0.000	0.000	0.000	0.040
A’U’	0.020	0.020	0.000	0.000
A’C’	0.020	0.000	0.020	0.000
A’G’	0.020	0.000	0.000	0.020
U’C’	0.000	0.020	0.020	0.000
U’G’	0.000	0.020	0.000	0.020
C’G’	0.000	0.000	0.020	0.020
A’U’C’	0.013	0.013	0.013	0.000
A’U’G’	0.013	0.013	0.000	0.013
A’C’G’	0.013	0.000	0.013	0.013
U’C’G’	0.000	0.013	0.013	0.013
A’U’C’G’	0.010	0.010	0.010	0.010

Notes: Adenosine (A’); uridine (U’); cytidine (C’); guanosine (G’). Based on economic efficacy and the previous studies of our research team, the concentration of nucleosides added in this experiment was determined as 0.04% (*w*/*v*).

## Data Availability

The original contributions presented in the study are included in the article, further inquiries can be directed to the corresponding author. due to (specify the reason for the restriction).

## References

[1] Zhou Y., Chu M.H., Ahmadi F., Agar O.T., Barrow C.J., Dunshea F.R., Suleria H.A.R. (2024). A Comprehensive Review on Phytochemical Profiling in Mushrooms: Occurrence, Biological Activities, Applications and Future Prospective. Food Rev. Int..

[2] Reis F.S., Barros L., Martins A., Ferreira I. (2012). Chemical composition and nutritional value of the most widely appreciated cultivated mushrooms: An inter-species comparative study. Food Chem. Toxicol..

[3] Liu Q., Kong W.L., Cui X., Hu S.J., Shi Z.W., Wu J., Zhang Y.T., Qiu L.Y. (2022). Dynamic succession of microbial compost communities and functions during *Pleurotus ostreatus* mushroom cropping on a short composting substrate. Front. Microbiol..

[4] Jafarpour M., Zand A.J., Dehdashtizadeh B., Eghbalsaied S. (2010). Evaluation of agricultural wastes and food supplements usage on growth characteristics of *Pleurotus ostreatus*. Afr. J. Agric. Res..

[5] Bhattacharjya D.K., Paul R.K., Miah M.N.A., Ahmed K.U. (2015). Comparative Study on Nutritional Composition of Oyster Mushroom (*Pleurotus ostreatus* Fr.) Cultivated on Different Sawdust Substrates. Biores. Commun..

[6] Pardo-Giménez A., Catalán L., Carrasco J., lvarez-Ortí M., Pardo J.E. (2015). Effect of supplementing crop substrate with defatted pistachio meal on *Agaricus bisporus* and *Pleurotus ostreatus* production. J. Sci. Food Agric..

[7] Coles P.S., Mcgiffen M.E., Xu H., Frutos M. (2024). Compost Filling Methods Affect Green Mold Disease Incidence in Commercial Mushrooms. Plant Dis..

[8] Kertesz M.A., Thai M. (2018). Compost bacteria and fungi that influence growth and development of *Agaricus bisporus* and other commercial mushrooms. Appl. Microbiol. Biotechnol..

[9] Carrasco J., Zied D.C., Pardo J.E., Preston G.M., Pardo-Giménez A. (2018). Supplementation in mushroom crops and its impact on yield and quality. AMB Express.

[10] Ding T., Song G., Liu X., Xu M., Li Y. (2021). Nucleotides as optimal candidates for essential nutrients in living organisms: A review. J. Funct. Foods.

[11] Claus-Peter W., Marco H. (2024). Nucleotides and nucleotide derivatives as signal molecules in plants. J. Exp. Bot..

[12] Zhang F., Klebansky B., Fine R.M., Xu H., Pronin A., Liu H., Tachdjian C., Li X. (2008). Molecular mechanism for the umami taste synergism. Proc. Natl. Acad. Sci. USA.

[13] Brock M. (2009). Fungal metabolism in host niches. Curr. Opin. Microbiol..

[14] Fox E.M., Howlett B.J. (2008). Secondary metabolism: Regulation and role in fungal biology. Curr. Opin. Microbiol..

[15] Economou C.N., Diamantopoulou P.A., Philippoussis A.N. (2017). Valorization of spent oyster mushroom substrate and laccase recovery through successive solid state cultivation of *Pleurotus*, *Ganoderma*, and *Lentinula strains*. Appl. Microbiol. Biotechnol..

[16] Morais A.R.C., Zhang J., Dong H., Otto W.G., Mokomele T., Hodge D., Balan V., Dale B.E., Lukasik R.M., Sousa L.D. (2022). Development of an ammonia pretreatment that creates synergies between biorefineries and advanced biomass logistics models. Green Chem..

[17] Sandhya C., Sumantha A., Szakacs G., Pandey A. (2005). Comparative evaluation of neutral protease production by *Aspergillus oryzae* in submerged and solid-state fermentation. Process Biochem..

[18] Masuko T., Minami A., Iwasaki N., Majima T., Nishimura S.I., Lee Y.C. (2005). Carbohydrate analysis by a phenol-sulfuric acid method in microplate format. Anal. Biochem..

[19] Liu Q., Zheng X., Du R., Shao Y., Wen Q., Shen X., Wang F., Qi Y., Shen J., Hu Y. (2024). Enrichment characteristics of Cd and Hg and regulation of heavy metal transporter signaling in *Pleurotus ostreatus*. Sci. Total Environ..

[20] AOAC (1996). AOAC Official Method 991.43. Total, Soluble, and Insoluble Dietary Fiber in Foods.

[21] Zhang Y.F., Zhu J., Zou Y., Ye Z., Guo L., Zheng Q. (2024). Insoluble dietary fiber from five commercially cultivated edible mushrooms: Structural, physiochemical and functional properties. Food Biosci..

[22] Guan A., Wang M., Gong Y., Huang T., Du Y., Zong S. (2025). Optimization of selenium biofortification by liquid fermentation based on 2,4-dichlorophenoxyacetic acid and its effect on nutritional value of *Pleurotus ostreatus*. J. Food Compos. Anal..

[23] Song F., Su D., Shen L., Qiu J., Keyhani N.O., Wang C. (2022). Influence of selenium on the mycelia of the shaggy bracket fungus, *Inonotus hispidus*. J. Sci. Food Agric..

[24] Singh U., Sharma S. (2022). Impact of bioaccumulated selenium on nutraceutical properties and volatile compounds in submerged fermented *Pleurotus eryngii* mycelia. J. Food Process. Preserv..

[25] Feng P., Gao M., Burgher A., Zhou T.H., Pramuk K. (2016). A nine-country study of the protein content and amino acid composition of mature human milk. Food Nutr. Res..

[26] Garcia A.D., Chavez J.L., Mechref Y. (2013). Sugar nucleotide quantification using multiple reaction monitoring liquid chromatography/tandem mass spectrometry. Rapid Commun. Mass Spectrom..

[27] Yu C.X., Dong Q., Chen M.J., Zhao R.H., Zha L., Zhao Y., Zhang M.K., Zhang B.S., Ma A.M. (2023). The Effect of Mushroom Dietary Fiber on the Gut Microbiota and Related Health Benefits: A Review. J. Fungi.

[28] Yang R., Dong S.J., Luo J.H., Ma F.F., Jiang W.M., Han C.C. (2022). Research Progress on the Function and Application of Proteins of Edible and Medicinal Mushrooms: A Review. Int. J. Med. Mushrooms.

[29] Leong Y.K., Yang F.C., Chang J.S. (2021). Extraction of polysaccharides from edible mushrooms: Emerging technologies and recent advances. Carbohydr. Polym..

[30] Širić I., Humar M., Kasap A., Kos I., Mioč B., Pohleven F. (2016). Heavy metal bioaccumulation by wild edible saprophytic and ectomycorrhizal mushrooms. Environ. Sci. Pollut. Res. Int..

[31] Tschowri N. (2016). Cyclic Dinucleotide-Controlled Regulatory Pathways in *Streptomyces* Species. J. Bacteriol..

[32] Dikec J., Olivier A., Bobée C., D’Angelo Y., Catellier R., David P., Filaine F., Herbert S., Lalanne C., Lalucque H. (2020). Hyphal network whole field imaging allows for accurate estimation of anastomosis rates and branching dynamics of the filamentous fungus Podospora anserina. Sci. Rep..

[33] Wiemer A.J., Wiemer D.F., Montchamp J.L. (2015). Prodrugs of Phosphonates and Phosphates: Crossing the Membrane Barrier. Phosphorus Chemistry I: Asymmetric Synthesis and Bioactive Compounds.

[34] Atila F. (2019). Lignocellulosic and proximate based compositional changes in substrates during cultivation of *Hericium erinaceus* mushroom. Sci. Hortic..

[35] Mikiashvili N., Wasser S.P., Nevo E., Elisashvili V. (2006). Effects of carbon and nitrogen sources on *Pleurotus ostreatus* ligninolytic enzyme activity. World J. Microbiol. Biotechnol..

[36] Levin D.E. (2011). Regulation of cell wall biogenesis in Saccharomyces cerevisiae: The cell wall integrity signaling pathway. Genetics.

[37] Li Y., Feng Y., Shang Y., Xu H., Xia R., Hou Z., Pan S., Li L., Bian Y., Zhu J. (2023). Sexual spores in edible mushroom: Bioactive components, discharge mechanisms and effects on fruiting bodies quality. Food Sci. Hum. Wellness.

[38] Liu L.W., Fang J.G., Liang X.F., He S. (2020). Nucleotide promotes feed intake and protein utilization via regulating the gene expression of feeding and nitrogen metabolism in juvenile Chinese perch (*Siniperca chuatsi*). Aquac. Nutr..

[39] Gameiro P.A., Laviolette L.A., Kelleher J.K., Iliopoulos O., Stephanopoulos G. (2013). Cofactor Balance by Nicotinamide Nucleotide Transhydrogenase (NNT) Coordinates Reductive Carboxylation and Glucose Catabolism in the Tricarboxylic Acid (TCA) Cycle. J. Biol. Chem..

[40] Salar-García M.J., Bernal V., Pastor J.M., Salvador M., Argandoña M., Nieto J.J., Vargas C., Cánovas M. (2017). Understanding the interplay of carbon and nitrogen supply for ectoines production and metabolic overflow in high density cultures of *Chromohalobacter salexigens*. Microb. Cell Fact..

[41] Zhou S., Zhang J., Ma F., Tang C., Tang Q., Zhang X. (2018). Investigation of lignocellulolytic enzymes during different growth phases of *Ganoderma lucidum* strain G0119 using genomic, transcriptomic and secretomic analyses. PLoS ONE.

[42] Elisashvili V., Kachlishvili E., Penninckx M.J. (2008). Lignocellulolytic enzymes profile during growth and fruiting of *Pleurotus ostreatus* on wheat straw and tree leaves. Acta Microbiol. Immunol. Hung..

[43] Wei Y., Zhuo F., Fan R., Liu F., Zhang H. (2013). Enhancing the Laccase Production and Laccase Gene Expression in the White-Rot Fungus Trametes velutina 5930 with Great Potential for Biotechnological Applications by Different Metal Ions and Aromatic Compounds. PLoS ONE.

[44] Masi A., Mach R.L., Mach-Aigner A.R. (2021). The pentose phosphate pathway in industrially relevant fungi: Crucial insights for bioprocessing. Appl. Microbiol. Biotechnol..

[45] Rauscher R., Würleitner E., Wacenovsky C., Aro N., Stricker A.R., Zeilinger S., Kubicek C.P., Penttilä M., Mach R.L. (2006). Transcriptional regulation of xyn1, encoding xylanase I, in *Hypocrea jecorina*. Eukaryot. Cell.

